# Screening the Hemostatic Active Fraction of *Artemisia annua* L. *In-vitro*

**Published:** 2011

**Authors:** Bochu Wang, Jing Sui, Zhengwen Yu, Liancai Zhu

**Affiliations:** *College of Bioengineering, Chongqing University, Chongqing, 400030, P.R.China.*

**Keywords:** *Artemisia annua L.*, Crude extract, Hemostasis, *In-vitro*, Plasma, Recalcification time

## Abstract

Artemisinin extracted from *Artemisia annua *L*. *is the best medicine with the highest efficiency, the most effective and the lowest toxicity in treating ague nowadays. At present, most studies focus on artemisinin and its derivatives, while the study and report about the other active components are rare. This paper purposed to further discover new indication of *Artemisia annua *L. connecting with the record of traditional medicine.

We screened the hemostatic active fraction of *Artemisia annua *L*. in-vitro *by plasma recalcification time (PRT). The crude extract and the extract of *n*-butanol were purified by polyamide, MCI, gel column in order. Determining the part of 20% methanol fraction after column chromatography of MCI gel is the hemostatic active fraction of *Artemisia annua *L. The shorten rate of clotting time are followed by: crude extract of *Artemisin annua *L. (8.51%); the *n*-butanol extract (14.89%); water eluting fraction after the extract of *n*-butanol was purificated by polyamide (22.11%); 20% methanol fraction after column chromatography of MCI gel (27.37%). It can provide experimental data for the clinical application of *Artemisia annua *L. which can be exploited as hemostatic. This topic has a certain academic value and potential prospects on the deep research of the *Artemisia annua *L. resource.

## Introduction

Blood loss, while minor in every day cuts and bruises, is one of the main causes of mortality. Hemorrhage threatens the life safety of patients and the wounded in trauma care and surgical intervention. Hemorrhage is the main reason in the causes of death in 48 h after trauma, which accounts for 80% in all trauma accident (1). Early control of hemorrhage remains the most effective strategy for treating combat casualties. Catastrophic blood loss often results in hemorrhagic shock as demonstrated in animal models (2-4), resembling human outcomes (5-7). Therefore development of compounds to improve hemostasis and save patient’s life in the trauma is of medical importance. 

A number of hemostatic agents have recently been deployed to the warfront that can be used to arrest bleeding before surgical control of the source (8). The ideal hemostatic agent would be easy to use, inexpensive, and rapidly available with no special storage requirements, non-immunogenic, versatile, and biologically inert with minimal side effects (9). Medicinal plants are used with in a context of a traditional medicine that confronts health and illness from an integral vision.

*Artemisia annua *L*. *is the only plant amedica which has been recognized to research and develope as the standards of western medicine research by the WHO in China. It grows wild in Europe and America, which is cropped on a large scale in China, Vietnam, Turkey, Iran, Afghanistan and Australia now (10). It is a famous herbs known for its highest efficiency, most effective and lowest toxicity in treating ague since artemisinin was extracted in 1977. It has been the subject of intensive phytochemical investigation following the discovery of the anti-malarial drug artemisinin (11). At present, most studies focus on artemisinin and its derivatives, while the study and report about the other active components are rare. Effect of *Artemisia annua *L*. *on hemostasis is known in traditional medicine (12-15). This paper purposed to further discover new indication *Artemisia annua *L*. *Screening the hemostatic active fraction of *Artemisia annua *L*. in-vitro *to expand multiple uses of resources of *Artemisia annua *L*. *

## Experimental

*Plant material*

*Artemisia annua *L*. *was collected from the GAP cultivation base in Youyang, Chongqing, southern China in september 2006 and dried. It was verificatied by researcher Luo Rongchang, and preserved in college of bioengineering, Chongqing University, P.R.China.

*Laboratory animal*

Japan purely rabbits utilized in the study (male/female 2.5-3.0 kg) purchased from the animal office in Daping, ChongQing, China.

*Main instrument and reagent*

AKTA Prime Plus bioseparation and purification system; polyamide column; MCI chromatographic column; rotatory evaporator RE-S2; LDZS2 equilibrium hydroextractor; thermostatic waterbath; ethanol; petroleum ether; acetic ether; *n*-butanol; sodium citrate. All chemicals were of analytical reagent grade and used as received which purchased from East of Sichuan Chemical Industry Group. Yunnan Baiyao purchased from Yunnan Baiyao Group Co., Ltd; doubly distilled water was used throughout. 

*Blood manipulation*

Blood samples, obtained freshly from normal rabbits. It was collected in plastic tubes and anticoagulated with sodium citrate 3.8% (1 part citrate: 9 parts blood) for the recalcification time test. Platelet-rich plasma (PRP) and platelet-poor plasma (PPP) were immediately prepared according to the standard procedures (16): PRP was obtained by collecting the supernatant plasma after the blood samples were centrifuged at 1000 rpm for 10 min, and PPP was obtained after the blood samples were centrifuged at 3000 rpm for 20 min. The plasma recalcification time test was performed soon after PRP and PPP were obtained. 

*Measurement of hemostatic activity*

The potential of plant extracts to hemostasis was assessed based on a procedure described by Kang Chen *et al*. (17). In the determination of the recalcification time of blood plasma, 0.1 mL of the plasma (PPP) was transferred to the detection extraction. The mixture was incubated at 37°C for 1 min, then recalcified by addition of 0.1 mL M/40 CaCl_2_ warmed to 37°C. A stopwatch was start as the calcium salt was added. The end of solidification reached since the plasma is fully concreting.

We gain the shortening rate of cloting time through the next mathematical formula:

$R=\frac{T_{1}-T_{2}}{T_{1}}\times 100$                     (Equation 1)

(R = shortening rate of clotting time; T_1_ = clotting time of control group; T_2_ = clotting time of medication administration team).

*Purification of hemostatic active fraction in Artemisia annua L.*

A quantity (40 kg) of *Artemisia annua *L*. *was extracted in a diffusion apparatus with 75% ethanol. The ethanolic extracts were filtered and evaporated to dryness under reduced pressure in a rotary evaporator (18).

We recorded the crude extract as O, and then extracted the crude extract by equal volume of petroleum ether (recorded as: A), ethyl acetate (recorded as: B), *n*-butanol (recorded as: C) one by one, the remaining part after the extraction of *n*-butanol (recorded as: D). The extracts then transferred to vials, kept at 4°C and examined for PRT. 

The extract of *n*-butanol was purificated by polyamide. Water eluting first, which was recorded as C_1_, and followed by different concentrations of ethanol(30%, 50%, 70%, 95%) to elute, which were record as C_2_, C_3_, C_4_, C_5_ respectively. 

C_1_ was segregated by column chromatography of MCI gel to gradient elute. The eluting solvent were water (recorded as: C_11_), and then eluted by 20% methanol, 60% methanol (recorded as: C_12_, C_13_ respectively). The conditions of the separation were: wave 365 nm; flow rate: 2 mL/ min; fraction size: 15 mL; pressure: 0.3 MPa. 

The preparation of physic liquor: 0.25 g extracts were dissolved in 50 mL doubly distilled water, and make sure it to be 0.5% concentration, then we filtering it by disposable filter. 

*Data analysis*

The potency of *artemisia annua L. *in stopping bleeding and cloting retraction was indicated as r-value (the shortening rate of clotting time to the negative control). The positive control is Yunnan BaiYao, which is the most widely used hemostatic agent in China. Repeat 6 times, significance analysis and statistical comparisons were performed by means of the originpro7.5. 

## Results

**Figure 1:**
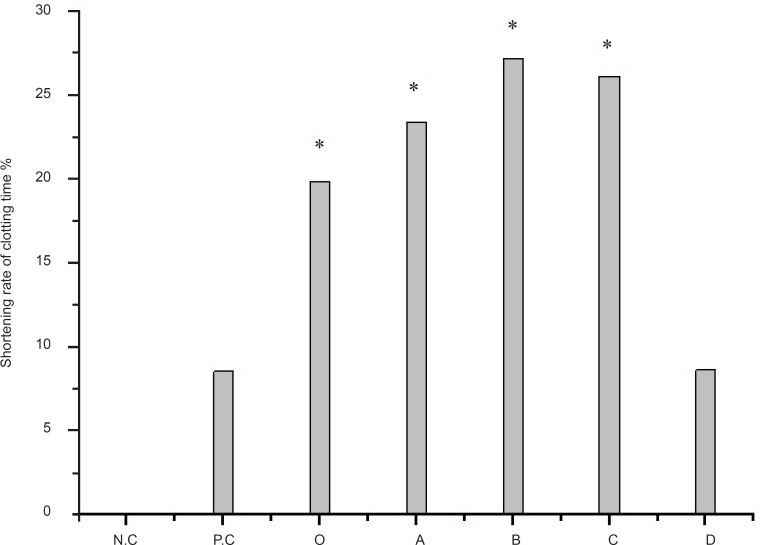
Determination of PRT on crude extract and the four extracts with organic solvents of *artemisia annua L. *p ≤ 0.05 Legend: N.C = negative control; P.C = positive control; O = crude extract of *artemisin annua L.*; A = the petroleum ether extract; B = the ethyl acetate extract; C = the *n-*butanol extract; D = the remaining part after the extraction of *n-*butanol

The hemostatic activity of the crude extract and the four extracts with organic solvents of *Artemisia annua *L*. *were provided in Figure 1. As displayed in Figure 1, the crude extract of *Artemisia annua *L*. *has the hemostatic activity, and the r-value is 19.85%, while the r-value of positive control is 8.54%. In Figure 1, because the ethyl acetate extract is not completely dissolved and there is granular which can accelerate the solidification of the plasma in the physic liquor, the r-value of ethyl acetate extract is a little high than the *n*-butanol extract. We also did another test called as clotting time adopting coagulation plate (ACP) (19), and the result showed that the r-value of *n*-butanol extract is higher. There was significant effect of the crude extract and *n*-butanol extract on PRT. 

**Figure 2:**
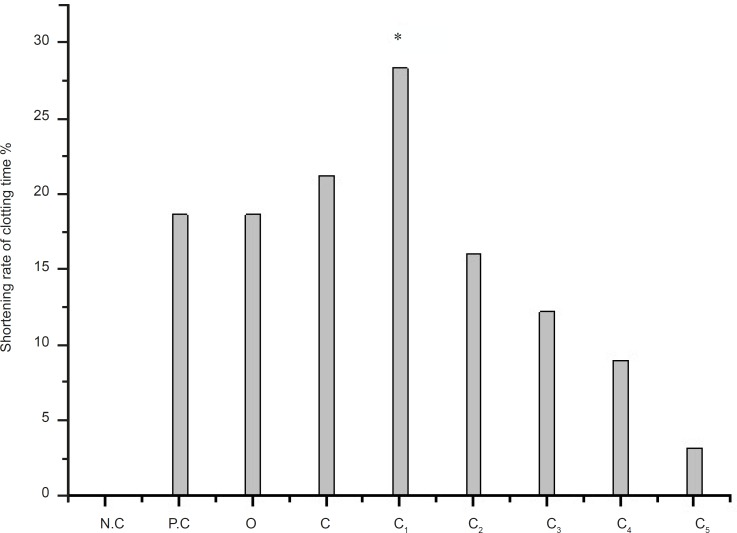
Determination of PRT on C (the *n-*butanol extract) from *artemisia annua L. *p ≤ 0.05 Legend: N.C = negative control; P.C = positive control; O = crude extract of *artemisin annua L.*; C = the *n-*butanol extract; C_1_ = water eluting fraction after the extract of *n-*butanol was purificated by polyamide; C_2 _= 30% ethanol fraction after the extract of *n-*butanol was purificated by polyamide; C_3_ = 50% ethanol fraction after the extract of *n-*butanol was purificated by polyamide; C_4_ = 70% ethanol fraction after the extract of *n-*butanol was purificated by polyamide; C_5_ = 95% ethanol fraction after the extract of *n-*butanol was purificated by polyamide

The effect of different eluting solvents from C (*n*-butanol extract) on PPT is shown in Figure 2. C_1_ has the higher r-value than the C. It is significant effect fraction after the *n*-butanol extract. The r-value is 28.36%, while the r-value of C is 21.27% and the r-value of positive control (recorded as: P.C ) is 18.68%. 

**Figure 3:**
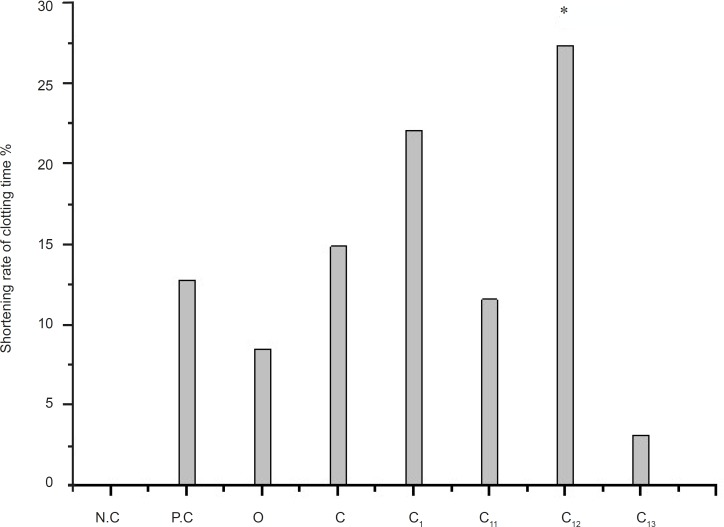
Determination of PRT on C_1_ from the *n-*butanol extract p ≤ 0.05 Legend: N.C = negative control; P.C = positive control; O = crude extract of *artemisin annua L.*; C = the *n-*butanol extract; C_1 _= water eluting fraction after the extract of *n-*butanol was purificated by polyamide; C_11_ = water eluting fraction after C_1_ was segregated by column chromatography of MCI gel; C_12_ = 20% methanol fraction after C_1_ was segregated by column chromatography of MCI gel; C_13_ = 60% methanol fraction after C_1_ was segregated by column chromatography of MCI gel

The effect of different eluting solvents from C_1_ (*n-*butanol extract) on PPT is shown in Figure 3. The r-value are followed by: O (8.51%); C (14.89%); C_1_(22.11%); C_12_(27.37%); P.C (12.77%). The rabbits we used in the research are different in every experiment, so the r-value of same part is slightly different in the three figures, while we determined the PRT of crude extract and the source of the activity site in every experiment. So the results are scientific and rationality. 

## Discussion

Hemostasis is important to save patient’s life. Plasma recovery-calcium time: decoagulant combined with the calcium of plasma to block up the coagulation process. The result shows that the *Artemisia annua *L*. *extract and C_12_ have obvious pro-coagulant effect *in-vitro*. The C_12 _from *Artemisia annua *L*. *might be the effective fraction. C_12_ is the part of 20% methanol fraction after column chromatography of MCI gel is the hemostatic active fraction of *Artemisia annua *L*. *The shorten rate of clotting time is (27.37%). 

Controlling hemorrhage will always remain a top priority in trauma care, and the development of materials to achieve this goal more effectively is of obvious benefit. In response to the changing combat and trauma casualty care, there has been an increase in efforts to develop better hemostatic agents. An ideal agent should be effective, easy to use, safe, logistically superior, and durable. 

With the current therapeutic condition of hemorrhage, an effective hemostatic drug is significant. Because *Artemisia annua *L*. *is sufficient, and the price is cheaper, screening the hemostatic active fraction of *Artemisia annua *L*. *to develop haemostat is superiority. Since *Artemisia annua *L*. *is derived from a plant source that the risk of disease transmission or allergic reaction is minimal. This also provides a readily available source to keep the overall cost of the product low. 

The present findings, although requiring confirmation by a larger trial, show that further research is needed with longer survival studies and direct comparisons to other hemostatic agents before widespread acceptance is prudent. 
